# Sox2 acts as a rheostat of epithelial to mesenchymal transition during neural crest development

**DOI:** 10.3389/fphys.2014.00345

**Published:** 2014-09-12

**Authors:** Nikolaos Mandalos, Muriel Rhinn, Zoraide Granchi, Ioannis Karampelas, Thimios Mitsiadis, Aris N. Economides, Pascal Dollé, Eumorphia Remboutsika

**Affiliations:** ^1^Stem Cell Biology Laboratory, Division of Molecular Biology and Genetics, Biomedical Sciences Research Centre “Alexander Fleming”Vari-Attica, Greece; ^2^Institut de Génétique et de Biologie Moléculaire et Cellulaire, INSERM, U964, CNRS, UMR7104, Université de StrasbourgIllkirch, France; ^3^Orofacial Development and Regeneration Unit, Faculty of Medicine, Institute of Oral Biology, University of Zurich, ZZMZurich, Switzerland; ^4^Department of Neurosurgery, University Hospitals Case Medical CenterCleveland, OH, USA; ^5^Regeneron PharmaceuticalsTarrytown, New York, NY, USA

**Keywords:** Sox genes, SoxB, neurocristopathies, craniofacial development, stem cells, neural progenitors, heterochrony

## Abstract

Precise control of self-renewal and differentiation of progenitor cells into the cranial neural crest (CNC) pool ensures proper head development, guided by signaling pathways such as BMPs, FGFs, Shh and Notch. Here, we show that murine Sox2 plays an essential role in controlling progenitor cell behavior during craniofacial development. A “*Co*nditional by *In*version” *Sox2* allele (*Sox2^COIN^*) has been employed to generate an epiblast ablation of Sox2 function (*Sox2^EpINV^*). *Sox2*^*EpINV*/+*(H)*^ haploinsufficient and conditional (*Sox2^EpINV/mosaic^*) mutant embryos proceed beyond gastrulation and die around E11. These mutant embryos exhibit severe anterior malformations, with hydrocephaly and frontonasal truncations, which could be attributed to the deregulation of CNC progenitor cells during their epithelial to mesenchymal transition. This irregularity results in an exacerbated and aberrant migration of Sox10^+^ NCC in the branchial arches and frontonasal process of the *Sox2* mutant embryos. These results suggest a novel role for Sox2 as a regulator of the epithelial to mesenchymal transitions (EMT) that are important for the cell flow in the developing head.

## Introduction

The head develops from anteriorly located cells of the epiblast. These cells form the neuroectoderm that gives rise to the brain and craniofacial structures stemming via epithelial to mesenchymal transitions (EMT). Balanced control between self-renewal of neural progenitors and their differentiation into cranial neural crest cells (NCCs) ensures proper head development. Cranial NCCs (CNCCs) arise from a NCC pool derived from the neural ectoderm, and give rise to most of the peripheral nervous system and craniofacial structures. NCCs are induced by interactions between the neuroectoderm and adjacent non-neural ectoderm (Dickinson et al., 1995; Selleck and Bronner-Fraser, 1995). These interactions are orchestrated by a combination of signaling molecules such as Wnt proteins, bone morphogenetic proteins (BMPs) (Liem et al., 1995), fibroblast growth factors (FGFs), retinoic acid and proteins of the Notch pathway (Labonne and Bronner-Fraser, 1998; Aybar et al., 2002; Aybar and Mayor, 2002; Christiansen et al., 2002; Endo et al., 2002; Garcia-Castro et al., 2002; Villanueva et al., 2002; Wu et al., 2003). NCCs delaminate from the dorsal neural tube, migrate along defined territories of the craniofacial complex and finally differentiate into many cell types, including neurons, glial cells, Schwann cells, melanocytes, and cells of the connective tissue (Ayer-Le Lievre and Le Douarin, 1982).

Neural crest (NC) development depends on the activation of NCC-specific genes at the neural plate border (Gammill and Bronner-Fraser, 2003; Heeg-Truesdell and Labonne, 2004; Huang and Saint-Jeannet, 2004). A number of these genes belong to the Sox (Sry HMG-box) family of transcription factors (subdivided into A-H groups) harboring an HMG-box as a DNA binding domain (Pevny and Lovell-Badge, 1997; Wegner, 1999; Wilson and Koopman, 2002; Bernard and Harley, 2010; Kamachi et al., 2013; Karnavas et al., 2013). Whereas all SoxE genes show expression in NCC progenitors at some point following NC induction, differences exist in the onset and sequence of events. Induction of NCC formation is triggered by the expression of SoxE genes (Gammill and Bronner-Fraser, 2002), such as *Sox8, Sox9*, and *Sox10* (Bowles et al., 2000). These genes are already expressed in all premigratory NCCs, while later their expression becomes restricted to distinct NCC-derived subpopulations (Stolt et al., 2004; Betancur et al., 2011). *Sox8* expression occurs before NCC migration from the neural tube, followed by *Sox9*, and shortly after *Sox10* (Cheng et al., 2000). *Sox9* expression overlaps with that of a number of NCC determinant genes, such as *FoxD3, Bmp4, cadherin 6b, Slug*, and *RhoB* (Liu and Jessell, 1998; Briscoe et al., 2003). BMP signaling drives the induction, formation, determination and migration of CNCCs (Nie et al., 2006). Cadherin 6b establishes the premigratory NCC domain in the neural tube (Taneyhill, 2008). Slug induces premigratory and migratory CNCC behavior (Del Barrio and Nieto, 2002). Foxd3 induces the segregation of NCC from the neural tube (Kos et al., 2001). Rho is involved in delamination of NCC from the dorsal neural tube (Rutishauser and Jessell, 1988; Cordero et al., 2011). As migration starts (Nakagawa and Takeichi, 1995; Jessel and Weiss, 1998), Slug, RhoB, N-cadherin, and cadherin 6b are down-regulated at the trunk level (Akitaya and Bronner-Fraser, 1992; Monier, 1995), while *FoxD3* expression persists in all migratory NCCs (Cheng et al., 2000; Dottori et al., 2001). On the other hand, *Sox10* persists only in the trunk NCC populations (Cheng et al., 2000; Remboutsika et al., 2011).

Amongst SoxB genes (*Sox1,2,3,14*, and *21*), *Sox2* has been reported to play a cell-autonomous role in NCC development (Pan and Schultz, 2011). *Sox2* is one of the early-activated genes in the developing neural plate (Graham et al., 2003; Wen et al., 2008; Hutton and Pevny, 2011), and its expression is reduced in the dorsal neural tube as NCCs segregate and migrate. Thereafter, *Sox2* expression is upregulated in a subset of cells that arrived at their final destination, and gradually becomes restricted to the glial sublineages (Aquino et al., 2006). *Sox2* prevents terminal differentiation of Schwann cells (Wakamatsu et al., 2004; Le et al., 2005) and represses the melanocyte fate (Laga et al., 2010). Ectopic expression of *Sox2* in embryonic ectoderm and neural plate explants reveals that Sox2 is sufficient to inhibit NCC formation both in chick and mouse embryos (Papanayotou et al., 2008; Remboutsika et al., 2011). It has been postulated that Sox2 counteracts NC development (Scherson et al., 1993; Placzek and Briscoe, 2005; Remboutsika et al., 2011), as the NCC marker *Slug* is only expressed at regions of low *Sox2* expression in premigratory and migratory NCCs (Wakamatsu et al., 2004). Despite evidence that points to an involvement for *Sox2* in multiple steps of NCC development in mouse embryo, its role is yet elusive.

Homozygous *Sox2*-null mouse embryos die around implantation (Avilion et al., 2003b; Mandalos et al., 2012). We have developed a conditional ablation strategy, using a “*Co*nditional by *In*version” *Sox2* allele (*Sox2^COIN^*) (Mandalos et al., 2012), in order to study the role of *Sox2* in epiblast-derived multipotent lineages. Here, we show that Sox2 plays an essential role in controlling the behavior of the progenitor cells during head development. EMT is affected in mutant embryos during CNCC development, resulting in hydrocephaly and frontonasal defects. These results suggest a novel role for *Sox2* as a rheostat of the EMTs that influence head development in mice.

## Materials and methods

### Experimental animals

Generation of *Sox2^COIN^* mice was described elsewhere (Mandalos et al., 2012). All animals were handled in strict accordance with good animal practice as defined by the Animals Act 160/03.05.1991 applicable in Greece, revised according to the 86/609/EEC/24.11.1986 EU directive regarding the proper care and use of laboratory animals and in accordance to the Hellenic License for Animal Experimentation at the BSRC “Alexander Fleming” (Prot. No. 767/28.02.07) issued after protocol approval by the Animal Research Committee of the BSRC “Alexander Fleming” (Prot. No. 2762/03.08.05).

### Genotyping

Tail, yolk sack or embryonic tissues were isolated and processed according to previously described methodology (Mandalos et al., 2012). PCR amplification conditions and primers used are described elsewhere (Mandalos et al., 2012).

### Embryo processing and histological analysis

For staging of the embryos, midday of the vaginal plug was considered as embryonic day 0.5 (E0.5). All embryos were harvested in cold 0.12 M phosphate-buffered saline (PBS, pH 7.5). For histological analysis, embryos were fixed with 10% formalin for 24 h at room temperature and then washed several times with PBS, placed in embedding cassettes and sectioned in a Leica RM2125RT microtome. Paraffin sections (10 μm) were stained with hematoxylin and eosin and mounted with xylene based mounting medium, according to standard procedures (Fischer et al., 2008; Cardiff et al., 2014).

Embryos were harvested and fixed with 4% paraformaldehyde (PFA) in PBS overnight at 4°C and thoroughly washed with PBS. Fixed embryos were incubated with 20% sucrose overnight at 4°C for cryoprotection before they were embedded with O.C.T. compound (VWR International), snap-frozen in dry ice and stored at −80°C. Sagittal sections were prepared using a Leica cryostat. Cryosections (10–12 μm) were collected on Superfrost Plus microscope slides (VWR International) and stored at −20°C before analysis. For *in situ* hybridization, embryos were fixed overnight in PFA 4% in PBS then rinsed three times in PBS/Tween (0.1%) followed by three times wash in methanol before storage at −20°. Conventional bright field and fluorescence microscopy was performed under a Leica MZ16FA stereoscope.

### RNA *in situ* hybridization

RNA probes for *in situ* hybridization reactions were prepared by *in vitro* transcription as previously described (Knuchel et al., 2000; Chotteau-Lelievre et al., 2006; Rhinn et al., 2011) The probes used were: *Hoxa2* and *Hoxb1*, kindly provided by Robb Krumlauf; *Sox2*, kindly provided by Robin Lovell-Badge; *Sox10*, kindly provided by Benoît de Crombrugghe. Whole-mount *in situ* hybridization (ISH) was performed using an Intavis InSituPro robot (detailed protocol available at http://empress.har.mrc.ac.uk/, gene expression section).

### Image analysis

Embryo dissections, conventional bright field and fluorescence microscopy were performed under a Leica MZ16FA stereoscope, equipped with a Leica 350 camera and Leica Software. Sections were photographed under a Leica M420 macroscope and DMLB/DM4000B microscopes equipped with Photometrics digital cameras and the CoolSnap imaging software (Roger Scientific).

## Results

### Epiblast deletion of *Sox2* results into lethality around E11

The epiblast is destined to derive all multipotent lineages in the mouse embryo. Previously generated, null alleles of *Sox2* (*Sox2^βgeo/βgeo^* and *Sox2*^*βgeo*2/*βgeo*2^) are responsible for an early embryonic lethal phenotype (Avilion et al., 2003a; Ekonomou et al., 2005), masking any subsequent role of *Sox2* in the generation of epiblast-derived multipotent lineages during development. Taking into account the challenges of the *Sox2* locus, in that its proximal promoter and coding region are entirely contained within a CpG island, and are also spanned by an overlapping transcript, *Sox2Ot*, which contains *mmu-miR1897* (Amaral et al., 2009; Shahryari et al., 2014), we developed a novel conditional by inversion *Sox2^COIN^* allele (Mandalos et al., 2012). The inverted COIN *Sox2* allele (*Sox2^INV^*) is functionally null (Mandalos et al., 2012), as *Sox2^INV/INV^* mutants recapitulate the phenotype of *Sox2^βgeo/βgeo^* (Avilion et al., 2003a), *Sox2*^*βgeo*2/*βgeo*2^ (Ekonomou et al., 2005) and *Sox2*^*EpINV/βgeo*2^ (Mandalos et al., 2012) embryos, which die around implantation.

**Figure 1:**
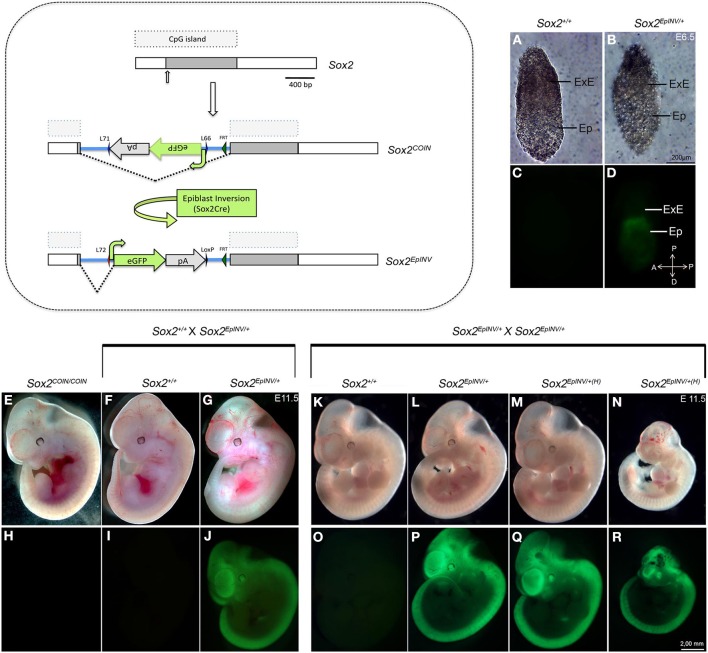
***Sox2* inversion in the epiblast leads to embryonic lethality at E11.5**. Generation of the epiblast-inverted *Sox2^EpINV^* allele. *Sox2*^*EpINV*/+^ embryos at E6.5 show normal morphology compared with *Sox2*^+/+^ control embryos. eGFP expression indicates that *Sox2* is highly expressed in the epiblast, but not in the extraembryonic tissue **(A–D)**. *Sox2*^*EpINV*/+^ embryos obtained from *Sox2*^*EpINV*/+^ × *Sox2*^+/+^ intercrosses are normal. At E11.5, *Sox2^COIN/COIN^* and *Sox2*^*EpINV*/+^ embryos are indistinguishable from *Sox2*^+/+^ littermates **(E–J)**. *Sox2*^*EpINV*/+^ intercrosses generate normal (*Sox2*^*EpINV*/+^) **(K,L,O,P)** and haploinsufficient (*Sox2*^*EpINV*/+*(H)*^) embryos **(M,N,Q,R)**. 50% of the haploinsufficient *Sox2*^*EpINV*/+*(H)*^ embryos have normal size **(M,Q)**, whereas the remaining ones have a smaller size **(N,R)** at E11.5. Both types of *Sox2*^*EpINV*/+*(H)*^ mutant embryos show evident defects in the head region **(M,N)**.

We generated epiblast inverted *Sox2*^*EpINV*/+^ mice, making use of the Tg(*Sox2*-CRE) mouse line that exerts efficient Cre-mediated recombination in the epiblast, but not in extraembryonic tissues (Hayashi et al., 2002a,b, 2003; Vincent and Robertson, 2003). Excision of the floxed sequences by Tg(*Sox2*-CRE) mice efficiently results in the visualization of eGFP in the epiblast at E6.5 (Figures 1A–D).

As *Sox2* is known to function as a cell fate determinant (Kondo and Raff, 2004; Yamaguchi et al., 2011; Karnavas et al., 2013), we harvested embryos from various intercrosses at the onset of organogenesis (E11.5). Initially, we performed *Sox2*^*EpINV*/+^ x *Sox2*^+/+^ intercrosses and found that heterozygous E11.5 *Sox2*^*EpINV*/+^ embryos do not show any obvious abnormalities and are indistinguishable from control *Sox2^COIN/COIN^* and *Sox2*^+/+^ littermates (Figures 1E–G). E11.5 *Sox2*^*EpINV*/+^ embryos express eGFP precisely in the areas where *Sox2* is expressed (Avilion et al., 2003a; Mandalos et al., 2012) (Figures 1H–J). *Sox2*^*EpINV*/+^ adult mice are fertile, feed normally, have normal body weight and normal lifespan, whilst *Sox2*^*INV*/+^ adult male exhibit no infertility problems, as some *Sox2^βgeo/+^* mice reportedly have (Avilion et al., 2003b). It is tempting to suggest that infertility problems in *Sox2*^*βgeo*2/+^ and *Sox2*^*βgeo*2/+^ mice could arise from the removal of regulatory regions due to the design of the mutants, while in *Sox2*^*EpINV*/+^ mice the whole sequence of the locus remains intact after inversion (Mandalos et al., 2012).

### Epiblast deletion of *Sox2* results into hydrocephaly and craniofacial defects

**Figure 2:**
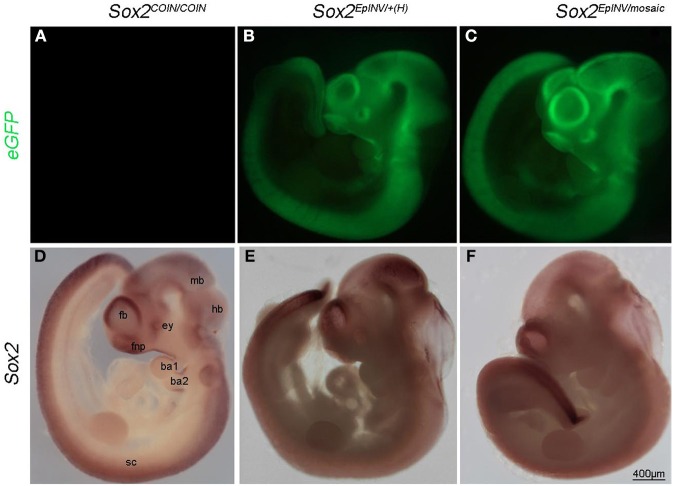
**Sox2 loss leads to multiple developmental defects**. eGFP expression recapitulates the expression of *Sox2* in *Sox2^EpINV/mosaic^* mutant **(A–C)**. Whole mount *in situ* hybridization shows down-regulation of *Sox2* in the hindbrain (hb), midbrain (mb) and forebrain (fb) regions, in the frontonasal process (fnp), the eye (ey), the surface ectoderm of branchial arches 1 and 2 (ba1, ba2), and the spinal cord (sc) but not the tail tip, of E10.5 *Sox2*^*EpINV*/+*(H)*^ and *Sox2^EpINV/mosaic^* embryos. These embryos show increased translucency mostly visible at the level of the hindbrain (hb); forebrain (fb) and midbrain (mb). Ventricles are also enlarged and frontonasal truncations are evident **(D–F)**.

We then proceeded to *Sox2*^*EpINV*/+^ intercrosses and harvested the embryos again at E11.5 (Table 1). Normally, if all *Sox2*^*EpINV*/+^ and *Sox2^EpINV/EpINV^* embryos would survive, one would expect that 75% of the embryos would be eGFP-positive (eGFP^+^). However, we observed that only 48.8% of the embryos harvested were eGFP^+^, suggesting that as previously observed (Mandalos et al., 2012), the *Sox2^EpINV/EpINV^* die in the deciduas at an early stage (Table 1). Half of the harvested eGFP^+^ heterozygote embryos had normal phenotypes *Sox2*^*EpINV*/+^ (Figures 1L,P), while the remaining ones represented haploinsufficient *Sox2*^*EpINV*/+^ (*Sox2*^*EpINV*/+(H)^) mutants (Figures 1Q,R). The *Sox2*^*EpINV*/+(H)^ observed embryonic phenotypes fall in two categories: (a) *Sox2*^*INV*/+(H)^ embryos with a similar size to *Sox2*^+/+^ and *Sox2*^*INV*/+^ embryos (Figures 1M,Q) and (b) *Sox2*^*INV*/+(H)^ embryos with a significantly reduced size (Figures 1N,R) compared to *Sox2*^+/+^ (Figures 1K,O) and *Sox2*^*EpINV*/+^ littermates (Figures 1L,P). *Sox2*^*EpINV*/+(H)^ embryos exhibit heart defects, hemorrhage, bulging fourth ventricular roof, and severe craniofacial defects (Figure 1M). We harvested litters at E12.5 and E15.5 derived from *Sox2*^*EpINV*/+^ intercrosses and found only *Sox2*^+/+^ and *Sox2*^*EpINV*/+^ normal embryos, suggesting that *Sox2*^*EpINV*/+(H)^ embryos die at around E11. Phenotypic differences among heterozygote *Sox2*^*EpINV*/+^ and *Sox2*^*EpINV*/+*(H)*^ littermates, derived from *Sox2*^*EpINV*/+^ intercrosses confirm that there is a *Sox2* expression threshold, below which phenotypic abnormalities appear during embryogenesis. To generate conditional epiblast-inverted *Sox2* mutants, we performed *Sox2^COIN/COIN^* to *Sox2*^*EpINV*/+^; Tg(*Sox2*CRE) intercrosses and named these conditional mutant embryos *Sox2^EpINV/mosaic^*. We could harvest *Sox2^EpINV/mosaic^* embryos from E8.5 to E11.5, but not beyond E11.5, indicating that these mutants, similarly to *Sox2*^*EpINV*/+(H)^ mutants, die around E11.5. *Sox2^EpINV/mosaic^* embryos exhibited similar, albeit more severe, abnormalities compared to *Sox2*^*EpINV*/+(H)^ embryos (Figures 2A–F).

**Table 1:** **Analysis of progeny from *Sox2*^*EpINV*/+^ × *Sox2*^*EpINV*/+^ intercrosses^#^**.

Genotypic distribution obtained at E11.5^*^						
**Total**	**Dead**	**Live**	***Sox2*^*EpINV*/+^**	***Sox2*^*EpINV*/+(*H*)^**	***Sox2*^*EpINV/EpINV*^**	***Sox2*^+/+^**
43	11 (25.6%)	32 (74.4%)	11 (25.6%)	10 (23.2)^**^	0 (0%)	11 (25.6%)

# *Data collected from mice in C57BL6 background*.

* *Genotypes were assessed by PCR either from tail biopsies or from embryonic yolk sac or whole embryos*.

** *50% of Sox2^INV/+ (H)^ (11.6% of the total number of embryos) from Sox2^INV/+^ × Sox2^INV/+^ intercrosses have a smaller size (Figures 1N,R)*.

To examine whether a regional loss of *Sox2* expression was responsible for those defects, we analyzed *Sox2* expression by RNA *in situ* hybridization (Figures 2D–F). *Sox2* was absent throughout the spinal cord (sc) in E10.5 mutants, with an exception of the tail tip of both *Sox2*^*EpINV*/+^ and *Sox2^EpINV/mosaic^* mutants (Figures 2E,F). However, there were no obvious morphological defects in the SC at E10.5, suggesting that down-regulation of *Sox2* could be rescued due to the functional redundancy of *Sox2* with other SoxB genes, namely *Sox1* and *Sox3* (Uwanogho et al., 1995; Rex et al., 1997; Wood and Episkopou, 1999; Archer et al., 2011; Elkouris et al., 2011). We observed a down-regulation of *Sox2* in the frontonasal process (fnp), the forebrain region (fb), the midbrain (mb) and the hindbrain (hb), the eye (Hever et al., 2006) and the otocyst (ot) (Kiernan et al., 2005; Hume et al., 2007; Pan et al., 2013) of *Sox2*^*EpINV*/+*(H)*^ and *Sox2^EpINV/mosaic^* mutant embryos. Morphologically, there was a distortion of the eye, an enlargement of the brain ventricles, and marked translucency in the hindbrain region.

**Figure 3:**
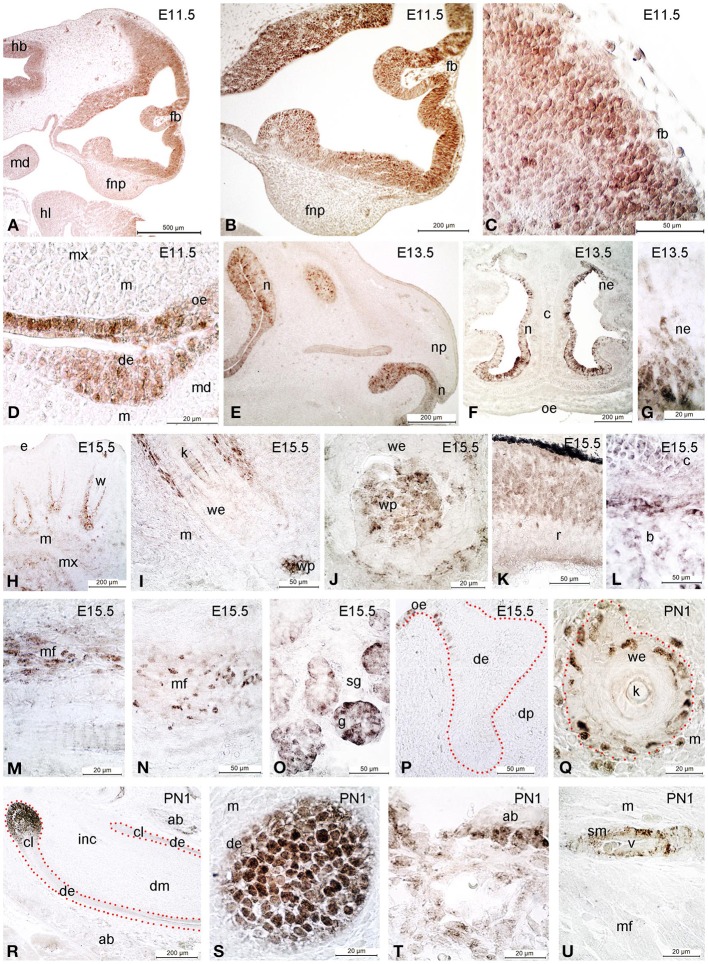
**Immunohistochemical analysis of Sox2 expression in developing craniofacial structures**. At E11.5 Sox2 is detected in hindbrain (hb) and forebrain (fb), as well as in the oral epithelium (oe) **(A–D)**. At E13.5, expression appears in the nasal epithelium (ne) **(E–G)**. At E15.5 Sox2 is expressed in the dermis surrounding the developing whiskers (w), and in their dermal papilla (wp) **(H–P)**. Expression is also detected in the retina (r, **K**), in developing bone (b) and cartilage (c, **L**), in developing muscle fibers (mf, **M,N**), and in salivary glands (sg, **O**). Noteworthy, the expression of Sox2 in the developing molar teeth is asymmetrical, being restricted to the connection between the tooth and the oral epithelium (oe) along the lingual side **(P)**. At postnatal day 1 (PN1), Sox2 expression is limited to some cells of the inner and outer root sheath of the whisker epithelium (we, the outer root sheath being delimited by red dots, **Q**) **(Q–U)**. Sox2 is furthermore expressed in the labial cervical loop (cl) of the incisor (inc) **(R,S)**, in the alveolar bone (ab) **(T,S)** and the vasculature **(U)**. de, dental epithelium; dm, dental mesenchyme; dp, dental papilla; fnp, frontonasal process; hl, hindlimb; m, mesenchyme; md, mandible; mx, maxilla; n, nose; np, nasal process; e, epithelium; g, glomerulae; k, keratinized part of the whisker; v, vessel; sm, smooth muscle; sg, salivary gland; c, cartilage; r, retina; f, follicle; mf, muscular fibers.

Several studies have shown that regulation of EMT during NCC development plays a crucial role for the normal development of the frontonasal region, including the palate and nasal cavities (Kang and Svoboda, 2005). We observed that Sox2 protein marks specific brain and craniofacial regions during their development (Figure 3). In E11.5 embryos, expression is seen in the hindbrain and forebrain neuroepithelium, and in the oral epithelium (Figures 3A–D). At E15.5, Sox2 is expressed in the dermis surrounding developing hair follicles and whiskers, including in their dermal papilla (Figures 3H–J). Sox2 is also detected in the epithelium of the retina (Figure 3K), in developing bone and cartilage (Figure 3L), in muscle fibers (Figures 3M,N) and the acinar structures of the salivary glands (Figure 3O). Furthermore, Sox2 asymmetrically marks the epithelium that connects the developing molar teeth with the oral epithelium (Figure 3P). At later post-natal stages Sox2 is expressed in the alveolar bone (ab) (Figure 3T) and in the incisor labial cervical loop (cl, Figures 3R,S). In the hair follicles the expression is confined to some cells of the inner and outer sheath (Figures 3Q,U).

**Figure 4:**
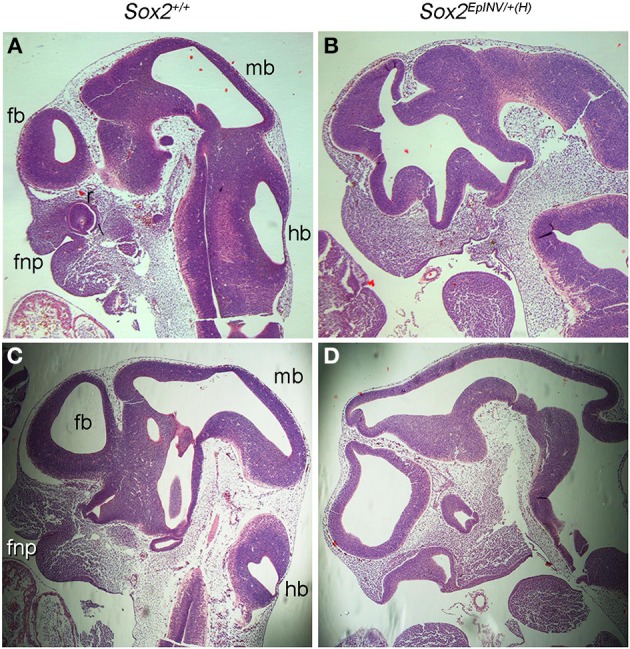
***Sox2*^*INV*/+*(H)*^ E11.5 embryos exhibit brain and craniofacial malformations**. Histological analysis of *Sox2*^+/+^ and *Sox2*^*EpINV*/+*(H)*^ embryos head structures **(A–D)**. Serial sagittal sections of E11.5 embryos stained with hematoxylin and eosin. *Sox2*^*EpINV*/+*(H)*^ embryos exhibit thinness of the ventricular wall and abnormal oral cavity formation enlarged forebrain and midbrain ventricles, while the frontonasal process (fnp) is severely reduced with an abnormal oral cavity **(B,D)** when compared *Sox2*^+/+^ littermates **(A,C)**.

To further analyze the craniofacial defects observed in *Sox2*^*EpINV*/+(H)^ embryos, we harvested E11.5 *Sox2*^*EpINV*/+*(H)*^ mutants and sectioned them for hematoxylin and eosin histological analysis (Figures 4A–D). Compared to *Sox2*^+/+^ normal embryos, we observed a reduction of the thickness of the neuroepithelial wall lining the telencephalic (fb), mesencephalic (mb) and rhombencephalic (hb) ventricles, defective frontonasal proccess and oral cavity formation, and dilated lumen of the optic stalk (Figures 4A–D). Thus, *Sox2* loss leads to severe brain and craniofacial defects.

### *Sox2* loss leads to down-regulation of *Hoxa2* in rhombomere 3, but not *Hoxb1* in rhombomere 4

**Figure 5:**
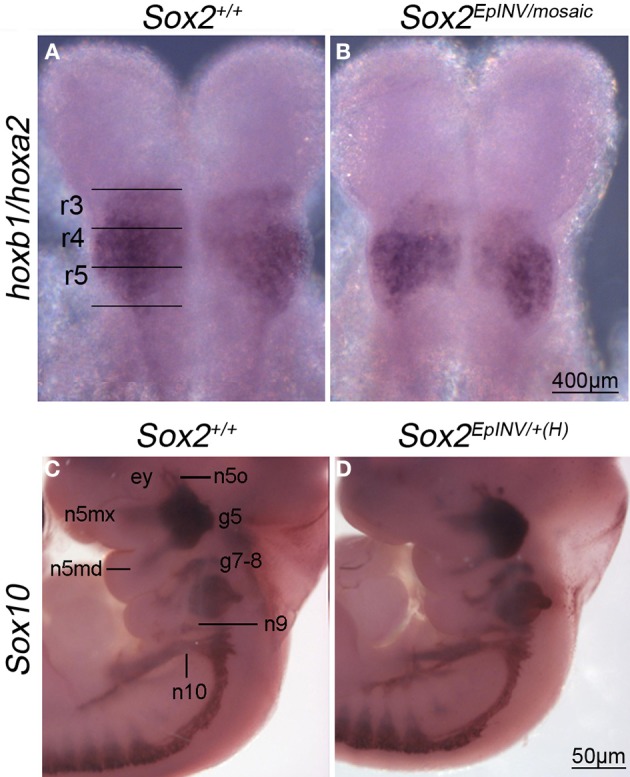
***Hoxa2* and *Hoxb1* expression in rhombomeres, and *Sox10* expression in developing cranial nerves and ganglia**. *In situ* hybridization for *Hoxa2* and *Hoxb1* at E8.5 reveals that *Hoxa2* is down-regulated in rhombomere 3 of a *Sox2^INV/mosaic^* embryo, but not in rhombomere 5 **(A,B)**. *Hoxb1* is normally expressed in r4 in *Sox2^INV/mosaic^* mutants **(A,B)**. *Sox10*^+^ nerves form normally in *Sox2*^*EpINV*/+*(H)*^ embryos. *In situ* hybridization reveals that *Sox10* expression is not affected in branchial arches 1 and 2 (ba1, ba2) in developing cranial nerves and ganglia (n10, n9, n5mx, n5md, g7-8, g5, n5o) of *Sox2^EpINV/mosaic^* mutants compared with *Sox2*^+/+^ control embryos **(C,D)**. ey, eye.

In order to find out whether *Sox2* loss of function disrupts the *Hox* code in hindbrain, we analyzed the expression of *Hoxa2* and *Hoxb1* at E8.5 in *Sox2*^+/+^ and *Sox2^EpINV/mosaic^* embryos, using whole mount *in situ* hybridization (Figures 5A,B). We observed that *Hoxa2* is down-regulated in rhombomere (r)3, but not in r5 of *Sox2^EpINV/mosaic^* mutant embryos, underscoring a specific role for *Sox2* in the regulation of *Hoxa2* in r3 at E8.5 (Figures 5A,B). To find out whether *Sox2* loss of function affects the facial innervation programme, we analyzed the expression of *Hoxb1* in *Sox2*^+/+^ and *Sox2^EpINV/mosaic^* embryos, and found that *Hoxb1* expression was unaffected both in r4 (Figures 5A,B) and in the spinal cord (data not shown) of E8.5 *Sox2^EpINV/mosaic^* embryos. Thus, our results do not support previous reports on *Sox2* involvement in the regulation of *Hoxb1 in vitro*, at least with regard to E8.5 hindbrain and spinal cord regions. As predicted from these observations, when cranial nerves were stained for *Sox10* expression at E11.5 in mutant embryos (Figures 5C,D), we found that *Sox2* loss of function does not affect the formation of *Sox10*^+^ ganglia of the spinal accessory, vagus (n10), glossopharyngeal (n9), and branches of facial (n7) nerves (Figures 5C,D).

### *Sox2* fine-tunes the flow of migrating *Sox10*^+^ NCCs

**Figure 6:**
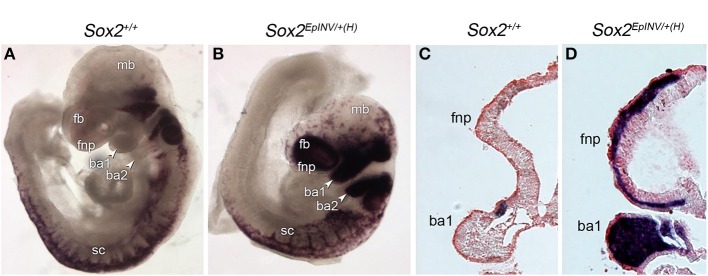
**Sox2 regulates the flow of *Sox10*^+^ NCC**. NCC formation and migration is exacerbated in E9.5 *Sox2*^*INV*/+*(H)*^ mouse embryos, as observed by the *Sox10 in situ* hybridization pattern in branchial arches 1 and 2 (ba1, ba2), and in the frontonasal (fnp) area of *Sox2*^*INV*/+*(H)*^ embryos when compared to *Sox2*^+/+^ embryos **(A,B)**. Coronal sections of E9.5 *Sox2*^*INV*/+*(H)*^ and *Sox2*^+/+^ embryos. Enlargement of the fourth ventricle and the mandibular component of the ba1 is observed. Exacerbated numbers of Sox10+ cells are observed in cranial and trunk regions, in the frontonasal region and throughout ba1 **(C,D)**. fb, forebrain; fnp, frontonasal process; mb, midbrain; sc, spinal cord; h, heart.

To investigate the fate of NCCs that may under to craniofacial abnormalities observed in *Sox2* mutants, we analyzed *Sox10* expression in *Sox2*^+/+^ and *Sox2*^*EpINV*/+(H)^ embryos at E9.5, at an embryonic stage in which *Sox10*^+^ NCCs are migrating along the lateral surface of the neural tube in wild-type embryos. We found that *Sox10*^+^ cells expressing high levels of *Sox10* are heterotopically present in the frontonasal region and in the branchial arches (ba) 1-2 of *Sox2*^*EpINV*/+*(H)*^ mutants (Figure 5A). Thus, down-regulation of *Sox2* causes a dramatic up-regulation of *Sox10* expression and an outflow of Sox10^+^ cells in the hindbrain (hb) and branchial areas of mutant embryos. Furthermore, we observed Sox10^+^ cells in *Sox2*^*EpINV*/+*(H)*^ mutant embryos in frontonasal areas, where *Sox10* is normally not expressed, while migrating CNCC do not express *Sox10* at this embryonic stage in *Sox2*^+/+^ control embryos (Figures 6A–D). Thus, *Sox2* loss disrupts severely the CNCC development.

## Discussion

EMT plays a crucial role in the development of the embryonic head (Mitsiadis, 2011). CNCCs undergo EMT and individual cells delaminate from the lateral ridges of the dorsal neural tube and migrate to the craniofacial area (Kouskoura et al., 2011) to form the frontonasal, maxillary and mandibular processes (Bronner-Fraser, 2002). Amongst genes of the SoxB1 group (*Sox1-3*), which are predominantly expressed in the developing central nervous system (CNS) (Collignon et al., 1996; Wood and Episkopou, 1999), *Sox3* activity is required for pharyngeal segmentation and for the pharyngeal epithelium to proceed toward craniofacial morphogenesis (Rizzoti and Lovell-Badge, 2007). On the other hand, *Sox2* activity has been implicated in processes that counteract NCC development (Hutton and Pevny, 2011; Remboutsika et al., 2011; Cimadamore et al., 2012) and could affect the generation of NCC progeny, as observed in differentiation experiments of human ES cells (ESC)-derived NCCs into sensory neurons *in vitro* (Cimadamore et al., 2012). Thus, the severity of defects observed in the developing brain and facial structures of *Sox2*^*EpINV*/+*(H)*^ and *Sox2^EpINV/mosaic^* mutants underline a Sox2 dosage-dependent role in the development of both the head and craniofacial areas.

*Sox2*^*EpINV*/+*(H)*^ and *Sox2^EpINV/mosaic^* embryos suffer from ventriculomegaly, which in turn leads to the accumulation of increased amounts of cerebrospinal fluid (CSF) in the brain. Hydrocephaly involves both dilated ventricular system and increased intracranial pressure. Every reduction in the thickness of the ventricular wall invevitably will result in dilation of the ventricular system (hydrocephalus ex vacuo). It is difficult to establish cause and effect relationship in this reciprocal setting, however abnormal brain wall development will definitely lead to morphologically enlarged ventricular system. The indication that the ventricular system is enlarged could not in itself be considered proof of hydrocephaly, because there is thickness of the wall, thus pointing to ventriculomegaly only (Deveale et al., 2013). Likewise, reduction in the diameter of the aqueduct and abnormal periaqueductal region development does not prove etiological relationship toward hydrocephalus causation (Lee et al., 2012). In adult animals, hydrocephaly involves also defects in choroid plexus, but as this is in very early stages in development at the embryonic period we examined, the contribution of CSF overproduction in hydrocephalus causation is hard to assess (Mizusawa, 1997). *Sox2*^*EpINV*/+*(H)*^ and *Sox2^EpINV/mosaic^* mutants display developmental defects both in ventricular system/wall formation, as well as in the oral cavity morphology that could contribute to early pathoanatomical events resulting in hydrocephalus and craniofacial defects in humans (Panetta et al., 2008). Thus, disruption of Sox2 function in the embryonic head region could be an additional cause for the development of hydrocephalus later on in life.

*Hox* genes play an essential role in the development of craniofacial structures (Trainor and Krumlauf, 2000; Narita and Rijli, 2009; Tumpel et al., 2009; Di Bonito et al., 2013). At E8.5, *Hoxb1* is expressed in r4 and throughout the spinal cord region (Gavalas et al., 2003), where it is required for the specification of facial branchiomotor neuron progenitors that are programmed to innervate the facial muscles (Arenkiel et al., 2004). Despite the fact that *Sox2* has been shown to regulate *Hoxb1 in vitro* (Di Rocco et al., 2001; Williams et al., 2004; Lian et al., 2010), the expression of *Hoxb1* appeared to be unaffected both in r4 and in the spinal cord of E8.5 *Sox2^INV/mosaic^* embryos (Figure 4). Our results do not support previous reports on *Sox2* involvement in the regulation of *Hoxb1 in vitro*, at least with regard to E8.5 hindbrain and spinal cord regions. On the other hand, *Hoxa2* appears to be down-regulated in our mutants. At E8.5, *Hoxa2* has a limit of expression in the rhombencephalic neural tube corresponding to r3 and r5 (Prince and Lumsden, 1994). It is not surprising that no effect was observed in *Hoxa2* expression in r5, as Sox2 is expressed along the hindbrain in all rhombomeres, but not in r5 (Wood and Episkopou, 1999). *Hoxa2*-null mutant embryos lack craniofacial and cartilage elements derived from the first and second branchial arch and die perinatally due to cleft palate (Vieille-Grosjean et al., 1997; Rijli et al., 1998; Trainor and Krumlauf, 2001; Santagati et al., 2005). Sox2 has been shown to interact *in vitro* with a *SoxB* DNA binding element (ACAAT motif) present in the enhancer of the *Hoxa2* gene and mutation of this motif reduces the expression of a *Hoxa2* reporter in electroporation experiments in chick embryo hindbrains (Tumpel et al., 2008). The reduction of *Hoxa2* expression in *Sox2^EpINV^* mutants indicates that *Sox2* controls an integral component of NCC morphogenetic program, which requires *Hoxa2* at discrete time points to pattern distinct derivatives in craniofacial structures (Santagati et al., 2005).

As neural progenitor cells differentiate into NCCs, a switch in expression from SoxB to SoxE genes becomes evident, with *Sox2* inactivated in the NCC progenitors, whereas *Sox9* and *Sox10* are activated in newly migrating trunk NCCs (Melton et al., 2004; Remboutsika et al., 2011). This is a necessary switch for the activation of the complex mechanism that generates NCCs (Wakamatsu et al., 2004). Amongst the SoxE genes, *Sox10* is required for the formation, maintenance of multipotency, specification and differentiation of NCCs (Kelsh, 2006). *Sox10* is the only *Sox*E gene that maintains its expression during migration of NCCs along the lateral surface of the neural tube (McKeown et al., 2005), except in the cranial region. *Sox10* mutations lead to several craniofacial abnormalities in humans, called neurocristopathies, including Waardenburg-Hirschsprung syndrome and peripheral neuropathies (Hoke, 2012). *Sox2* over-expression and Sox2^+^ neural stem cell transplantation experiments in avian and murine cranial neural tubes have demonstrated that Sox2 restricts neuroepithelial differentiation into CNCCs (Cheung and Briscoe, 2003; Remboutsika et al., 2011; Wahlbuhl et al., 2012). Thus, the exacerbation of *Sox10*^+^ migrating cells in the *Sox2^EpINV^* mutants may not be surprising. These observations point out that Sox2 could act to repress *Sox10* expression. However, any genetic interaction between Sox2 and Sox10 in neural progenitor or NCC progenitor cells is far from evident *in vivo* and *in vitro*. Whether Sox2 could influence the expression of Sox10 directly or indirectly by affecting levels of other SoxE genes such as *Sox8* and *Sox9* that contribute to the induction of *Sox10* in NCC progenitors, once NC-inducing signals are set (Taylor and Labonne, 2005; McCauley and Bronner-Fraser, 2006; Haldin and Labonne, 2010; Stolt and Wegner, 2010; Wahlbuhl et al., 2012), remains to be investigated. In the *Sox2^EpINV^* mutants, *Sox10* levels appear dramatically increased both in the branchial arches area and in the frontonasal area. *Sox10* over-expression has been shown to arrest the neuroepithelial and cranial mesenchymal cells in an undifferentiated state, causing a range of cell fate specification defects (Ahlstrom and Erickson, 2009). Neural progenitor cells, which over-express *Sox10* remain undifferentiated and fail to form neuronal, Schwann, or melanocyte cells (Stolt et al., 2008). Thus, it is tempting to suggest that the failure of the embryos to form the craniofacial region could, in part, be due to the failure of the cranial mesenchyme to proceed through development due to an aberrant and exacerbated population of *Sox10*^+^ cells in the frontonasal region.

**Figure 7:**
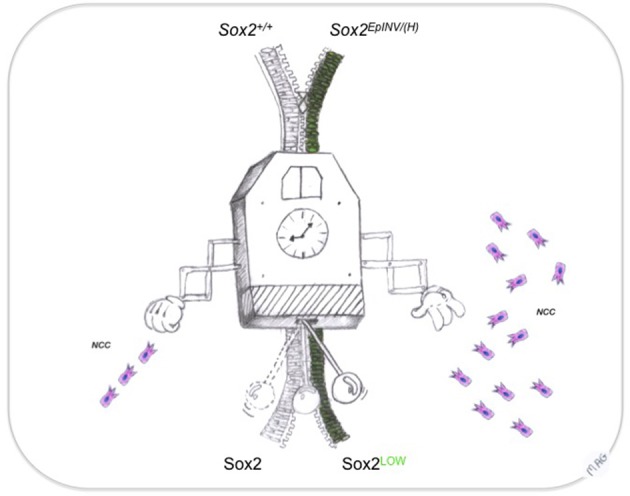
**Sox2: a rheostat of EMT transition during neural crest development**. Precise timing ensured by an extremely accurate developmental clock regulates the dynamics of the decisions to generate NCC from neural progenitors. Sox2 controls the flow of the EMT transition, leading to NCC migration in appropriate numbers at the appropriate regions in the head. This way, sequence (from neural progenitor to NCC) and genetic heterochrony (spatially and temporally controlled Sox10 expression), resulting into craniofacial malformations could be averted.

In recent years, the importance of NCCs as inducers of peripheral neural structures, craniofacial tissues and other peripheral mesodermal-derived structures has become evident (Trainor and Tam, 1995; Trainor et al., 2003; Hong and Saint-Jeannet, 2005; Cordero et al., 2011; Hagiwara et al., 2014). Defects in their development has been attributed to a failure and/or abnormal NCCs migration and differentiation (Bronner, 2012), resulting into the generation of neurocristopathies in humans (Etchevers et al., 2006) and expanding the most recent classification of neurocristopathies to an entire category of abnormal induction of non-neural NCC-derived peripheral structures of the body (Cossais et al., 2010). Recent evidence has shown that *Sox2* has been indirectly associated with defects that are characteristic of the CHARGE syndrome, a human neurocristopathy (Aramaki et al., 2007). CHARGE syndrome patients exhibited mutations in the *Chd7* gene (Vallaster et al., 2012), the product of which acts as a *Sox2* transcriptional cofactor (Engelen et al., 2011; Puc and Rosenfeld, 2011). Our results suggest a *Sox2* dosage-dependent mechanism acting during head development, with a specific role for Sox2 in the prevention of these CNCC-related pathologies. We propose that *Sox2* acts as a rheostat of EMT during CNCC development that influences cell fates involved in head development (Figure 7). These findings open novel avenues to target *Sox2* in a number of craniofacial malformations in humans.

### Conflict of interest statement

The authors declare that the research was conducted in the absence of any commercial or financial relationships that could be construed as a potential conflict of interest.
